# Akt phosphorylation on Thr308 but not on Ser473 correlates with Akt protein kinase activity in human non-small cell lung cancer

**DOI:** 10.1038/bjc.2011.132

**Published:** 2011-04-19

**Authors:** E E Vincent, D J E Elder, E C Thomas, L Phillips, C Morgan, J Pawade, M Sohail, M T May, M R Hetzel, J M Tavaré

**Affiliations:** 1 School of Biochemistry, Medical Sciences Building, University of Bristol, Bristol BS8 1TD, UK; 2 Department of Respiratory Medicine, Bristol Royal Infirmary, Bristol BS2 8HW, UK; 3 Division of Histopathology, Bristol Royal Infirmary, Marlborough Street, Bristol BS2 8HW, UK; 4 School of Social and Community Medicine, Canynge Hall, University of Bristol, 39 Whatley Road, Bristol BS8 2PS, UK

**Keywords:** protein kinase B, Akt, biomarker, tumour, phosphorylation

## Abstract

**Background:**

The activity of the protein kinase Akt is frequently dysregulated in cancer and is an important factor in the growth and survival of tumour cells. Akt activation involves the phosphorylation of two residues: threonine 308 (Thr308) in the activation loop and serine 473 (Ser473) in the C-terminal hydrophobic motif. Phosphorylation of Ser473 has been extensively studied in tumour samples as a correlate for Akt activity, yet the phosphorylation of Thr308 or of downstream Akt substrates is rarely assessed.

**Methods:**

The phosphorylation status of Thr308 and Ser473 was compared with that of three separate Akt substrates – PRAS40, TSC2 and TBC1D4 – in fresh frozen samples of early-stage human non-small cell lung cancer (NSCLC).

**Results:**

Akt Thr308 phosphorylation correlated with the phosphorylation of each Akt substrate tested, whereas Akt Ser473 phosphorylation did not correlate with the phosphorylation of any of the substrates examined.

**Conclusion:**

The phosphorylation of Thr308 is a more reliable biomarker for the protein kinase activity of Akt in tumour samples than Ser473. Any evaluation of the link between Akt phosphorylation or activity in tumour samples and the prediction or prognosis of disease should, therefore, focus on measuring the phosphorylation of Akt on Thr308 and/or at least one downstream Akt substrate, rather than Akt Ser473 phosphorylation alone.

The stimulation of cell growth and survival by numerous growth factors involves the activation of the phosphoinositide 3-kinase (PI3-kinase). This leads to the production of phosphoinositide(3,4,5)trisphosphate (PI(3,4,5)P_3_) and the consequent activation of the protein serine/threonine kinase Akt (also known as protein kinase B). The activation of Akt involves the phosphorylation of two residues: threonine 308 (Thr308) in the activation loop of the kinase by the protein kinase PDK1, and serine 473 (Ser473) in the hydrophobic motif by the mTORC2 complex (Alessi *et al*, 1997; Vanhaesebroeck and Alessi, 2000; Sarbassov *et al*, 2005). Activated Akt then phosphorylates numerous downstream substrates, including glycogen synthase kinase-3 (GSK3*α*/*β*), tuberous sclerosis complex-2 (TSC2), Foxo-family transcription factors, Bad, Par-4, PRAS40 and TBC1D4 (Manning and Cantley, 2007). It is these proteins that together provide potent growth, survival and metabolic signals to the cell (Hanada *et al*, 2004; Goswami *et al*, 2005).

The Akt pathway represents one of the most frequently dysregulated pathways found in human cancer (Altomare and Testa, 2005; Cheng *et al*, 2005) and is a critical mediator of the survival and proliferative effects of several oncogenes including mutant forms of the EGF receptor, Ras and PI3-kinase, as well as the Bcr-Abl fusion protein (Yuan and Cantley, 2008). The activity of the Akt pathway is counteracted by the PI(3,4,5)P_3_ phosphatase, PTEN, and mutations in or haploinsufficiency of PTEN are frequently observed in human tumours (Li *et al*, 1998; Cantley and Neel, 1999; Ramaswamy *et al*, 1999; Sun *et al*, 1999).

Because of the importance of the Akt pathway in tumourigenesis, Akt activity represents a potential biomarker for use in disease prognosis or in predicting tumour response to therapeutics. This biomarker may provide an indicator of sensitivity or resistance to small molecule therapeutics directed at upstream regulators such as the epidermal growth factor receptor (EGFR) or PI3-kinase, as well as Akt itself (Pal *et al*, 2010).

This leads to the question of how best to evaluate Akt activity as a biomarker. In the vast majority of studies on Akt in cancer, the measurement of phosphorylation on Ser473 has been used as an indicator of Akt activity. However, using Ser473 as a measure of Akt activity often leads to contradictory conclusions regarding whether Akt activity is prognostic for various cancers. For example, in non-small cell lung cancer (NSCLC) phosphorylation of Akt on Ser473 has in some studies been found to correlate with poor prognosis (David *et al*, 2004; Tang *et al*, 2006; Lim *et al*, 2007), whereas others have found that it has no prognostic significance (Mukohara *et al*, 2003; Tsao *et al*, 2003; Massion *et al*, 2004). Contradictory observations have also been found in other types of tumour; phosphorylation on Ser473 has been correlated with poor prognosis in breast and ovarian cancer (Perez-Tenorio and Stal, 2002; Dai *et al*, 2005) whereas other studies find this not to be the case (Slipicevic *et al*, 2005; Al-Bazz *et al*, 2009). Interpretation of such data is confounded by whether Akt phosphorylation on Ser473 is a reliable surrogate for Akt activity, which is ultimately what is important for the proliferation and survival of the tumour cells.

A more reliable *in vivo* biomarker of Akt activity may be the phosphorylation of Thr308, arguably the more important regulator of Akt activity (Vanhaesebroeck and Alessi, 2000). Although much more rarely investigated than Ser473 phosphorylation, there are reports that the phosphorylation of Thr308 correlates with poor survival in NSCLC (Tsurutani *et al*, 2006) and acute myeloid leukaemia (Gallay *et al*, 2009) and, interestingly, in both reports no such correlation was seen with Akt Ser473 phosphorylation. This suggests that the extent of Thr308 phosphorylation may be the better predictor of Akt activity.

However, the most reliable measure of Akt activity *in vivo* may be through the analysis of the phosphorylation of downstream targets of the kinase along with the phosphorylation of Akt itself. There are very few studies that have done this.

We wished to carry out such an analysis for NSCLC as it continues to be a leading cause of cancer death worldwide. In addition, studies relating Akt activity to clinical outcome in NSCLC are contradictory possibly because they rely almost solely on the phosphorylation of Ser473 as a marker. We have therefore undertaken a detailed and quantitative examination of the activation of Akt in normal and patient-matched tumour tissue from individuals with NSCLC. Herein we analyse the phosphorylation of Akt on Ser473 and Thr308 and correlate this with the activity of the kinase as determined by the phosphorylation of three well-characterised substrates: PRAS40 (Nascimento and Ouwens, 2009), TSC2 (Cai *et al*, 2006) and TBC1D4 (Kane *et al*, 2002).

## Materials and methods

### Materials

Rabbit polyclonal pAkt (Thr308), pAkt (Ser473), pTBC1D4 (Thr642), pPRAS40 (Thr246) and pTSC (Ser939) antibodies were purchased from Cell Signalling Technology (Boston, MA, USA). Mouse anti-F_1_-ATPase antibody was from Santa Cruz Biotechnology Inc. (Santa Cruz, CA, USA). Donkey horseradish peroxidase-conjugated anti-mouse IgG and anti-rabbit IgG antibodies were purchased from Jackson ImmunoResearch Laboratories (Bar Harbor, ME, USA).

### Patients and tissue samples

Patients with suspected lung cancer were identified from an elective thoracic surgery list at the Bristol Royal Infirmary, Bristol, UK. A Research Nurse contacted study subjects regarding participation and informed consent was given to use their tumour and matched normal lung tissue. The study was approved by the local research ethics committee (REC no: 07/Q2002/6, South West 4 REC).

The research nurse was present at thoracic surgery to collect the tissue, which was surplus to diagnostic requirements. This was separated into individual samples of tumour and normal tissues, which were immediately snap frozen in liquid nitrogen. A pathologist recorded the histology and stage of the tumour, and made an assessment of the percentage of tumour cells present in each tumour sample. Only those NSCLC tumour samples comprising at least 90% of tumour cells were included further in the study. All samples were stored at −80 °C until required for analysis.

The cohort had an average age of 64.5 years, 71% were male, 10% were non-smokers, 10% had accumulated 1–10 pack years, 27% 11–30 pack years and 53% >30 pack years (one pack year being defined as 20 manufactured cigarettes smoked per day for 1 year). Three patients had previously been exposed to asbestos. Following surgery, all tumours were confirmed to be stage I/IIa and histology showed that 53% were adenocarcinomas, 40% squamous cell carcinomas and 3% bronchiolalveloar carcinomas.

### Preparation of tissue lysates

Frozen tissue samples were homogenised using a Polytron homogeniser in 3 ml ice-cold extraction buffer (50 mM Tris pH 7.5, 120 mM NaCl, 1% (v/v) Nonidet-P40, 40 mM *β*-glycerophosphate, 1 mM benzamidine, 1 mM EDTA, 50 mM NaF, 10 mM Na_4_P_2_O_7_, 10 *μ*g ml^–1^ pepstatin, 10 *μ*g ml^–1^ antipain, 10 *μ*g ml^–1^ leupeptin, 5 mM Na_3_VO_4_ and 2 mM phenylmethylsulphonyl fluoride). Samples were pulsed for 10 s followed by a 30-s incubation on ice; a procedure repeated three times. Tissue lysates were rotated end-over-end at 4 °C for 30 min, and rested on ice for 15 min before centrifugation at 16 000 **g** for 15 min to remove insoluble material. Protein concentration was determined by BCA assay (Thermo Scientific, Rockford, IL, USA). Tissue lysates were stored at −80 °C.

### Western blot analysis

Lysates (15 *μ*g protein) were solubilised by boiling in 4 × Laemmli sample buffer and separated by SDS–PAGE using 4-12% gradient gels (Invitrogen Ltd, Paisley, UK). Proteins were transferred to polyvinylidene difluoride membranes (Millipore, Hertfordshire, UK). Membranes were blocked using 5% (w/v) bovine serum albumin in TBS-T (20 mM Tris pH 7.4, 137 mM NaCl, 0.1% (v/v) Tween-20) for 1 h, washed five times in TBS-T and then incubated with primary antibody (1 *μ*g ml^–1^) for 1 h in TBS-T containing 5% w/v bovine serum albumin. The membranes were again washed five times in TBS-T and then incubated with the appropriate secondary antibodies diluted in TBS-T for 1 h. Immunoblots were visualised using the enhanced chemiluminescence (ECL) detection system (Amersham Biosciences, Amersham, UK).

### Data and statistical analysis

Three separate samples of tumour tissue and three separate samples of normal tissue from an individual patient were subjected to western blotting and ECL detection. Normal and tumour protein samples from individual patients were separated on different gels and therefore the absolute levels of phosphorylation between patients were not compared. The extent of phosphorylation of each protein was quantified by densitometric scanning of the western blot (Quantity One Software, Bio-Rad, Hemel Hempstead, UK). Data are expressed as mean±s.e.m., and the strength of evidence for the difference in phosphorylation between the normal and tumour samples was determined by Kruskal–Wallis rank order test (*P*<0.05). Changes in phosphorylation in tumour samples relative to normal samples were coded with the values 0 (signifying an increase observed at *P*⩽0.05), 1 (decrease at *P*⩽0.05) or 2 (no change, i.e., *P*>0.05) in the tumour tissue relative to normal. The Spearman's distribution-free rank correlation test was applied to the data to test pair-wise correlations between the phosphorylation of Akt on Thr308 or Ser473 and the phosphorylation of the three Akt substrates PRAS40, TSC2 and TBC1D4. Quantitatively similar results were obtained when analysing the data using the Pearson's correlation coefficient test (data not shown).

## Results

The study cohort comprised 29 patients undergoing thoracic surgery for suspected lung cancer. Following resection from the patient, three samples of tumour were snap frozen in liquid nitrogen. Samples were confirmed by analysis of frozen sections to consist of at least 90% tumour tissue. Three samples of normal lung tissue were also taken from the resection margin surrounding the tumour and snap frozen. The use of patient-matched normal tissue allowed us to determine whether protein phosphorylation was increased, decreased or unchanged in the tumour relative to normal lung from that particular individual.

### Analysis of Akt phosphorylation on Thr308 and Ser473

Three separate samples of tumour and three samples of patient-matched normal tissue were lysed and subjected to SDS–PAGE followed by western blotting for the level of phosphorylation of Akt on Thr308 and Ser473, as well as the phosphorylation of the well-characterised downstream Akt substrates PRAS40 (on Thr246), TSC2 (on Ser939) and TBC1D4 (on Thr642).

**Figure 1:**
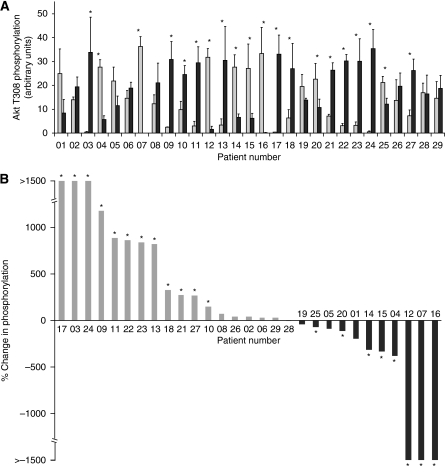
Phosphorylation of Akt on Thr308 in NSCLC tumour tissue in comparison with patient-matched normal lung tissue. Triplicate samples of lysate from patient-matched normal (N1–3) and tumour (T1–3) tissues were separated on SDS–PAGE gels. Phosphorylation of Akt on Thr308 was determined by western blotting with a pAkt-Thr308 antibody followed by quantitation by densitometric scanning. (**A**) Quantified data for all 29 patients. Each bar represents the average phosphorylation for normal (N1–3; light grey) or tumour (T1–3; black) tissue for each patient (mean±s.e.m.). The strength of evidence for a difference in phosphorylation between the normal and tumour samples was determined by Kruskal–Wallis test; ^*^P<0.05. (**B**) The percentage change in Akt-Thr308 phosphorylation in tumour samples in comparison with patient-matched normal tissue where patients are ranked in order of the extent of the percentage change in phosphorylation.

**Figure 2:**
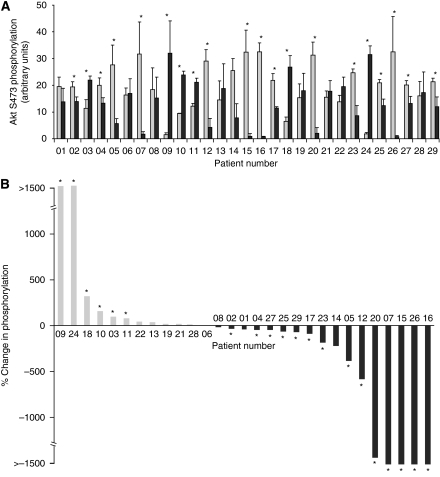
Phosphorylation of Akt on Ser473 in NSCLC tumour tissue in comparison with patient-matched normal lung tissue. Samples were western blotted and the data quantified exactly as described in Figure 1. (**A**) The extent of Ser473 phosphorylation in normal (N1–3; light grey) or tumour (T1–3; black) tissue for each patient (mean±s.e.m.; ^*^P<0.05). (**B**) The percentage changes in Akt Ser473 phosphorylation in tumour samples in comparison with patient-matched normal tissue with patients are ranked according to the percentage change in phosphorylation.

Figure 1A shows data compiled for the phosphorylation of Akt on Thr308 in all 29 tumour samples (dark grey bars) compared with samples of patient-matched normal tissue (light grey bars). In order to compare the data between patients, the data are reorganised in Figure 1B according to the magnitude of the fold change: from highest fold increase to highest fold decrease in tumour compared with normal tissue. Figure 2A and B shows a parallel analysis of the phosphorylation of Akt on Ser473.

Akt phosphorylation on Thr308 was increased in 12 out of 29 (41%) of tumours (at *P*<0.05; Kruskal–Wallis test) when compared with normal tissue. The magnitude of the change varied, but in four tumours this was in excess of a ten-fold increase. Akt Thr308 phosphorylation was decreased in 8 out of 29 (28%) of tumours (*P*<0.05); again the magnitude varied but in three tumours this was greater than a ten-fold decrease. In the remaining nine tumours, Akt Thr308 phosphorylation was apparently unchanged (i.e., *P*>0.05).

A similar analysis of the data for Akt phosphorylation on Ser473 showed that it was increased in 6 out of 29 tumours (21% *P*<0.05), decreased in 14 out of 29 tumours (48% *P*<0.05) and unchanged in the remaining 9 tumours, when compared with normal tissue.

The frequency of the increased phosphorylation was, therefore, less for Ser473 phosphorylation than we observed for Thr308, but conversely the decreases were more frequent. The reasons for the decreases observed are not known but may be the result of downregulation of Akt signalling in some tumours.

**Figure 3:**
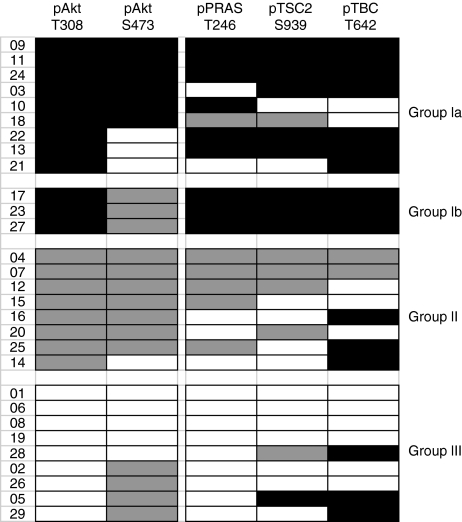
Examination of the correlation between Akt Thr308 and Ser473 phosphorylation and the phosphorylation of Akt substrates. Triplicate samples of normal and patient-matched tumour tissues were separated on SDS–PAGE gels. Phosphorylation of Akt (on Thr308 and Ser473) and three downstream substrate proteins (PRAS40-Thr246, TSC2-Ser939 and TBC1D4-Thr642) was determined by western blotting with phospho-specific antibodies. The data were quantified by densitometry and the strength of evidence of difference in phosphorylation between the normal and tumour samples was determined by Kruskal–Wallis test. Phosphorylation of each protein/site was then coded according to whether it increased (black), decreased (grey) at *P*⩽0.05 or remained unchanged (white; *P*>0.05) in the tumour samples relative to normal tissue. The coded data were then grouped according to the direction of change in Thr308 phosphorylation: patients in group I showed an increase, group 2 a decrease and group 3 no change. Group I were further subdivided according to the change in Akt Ser473 phosphorylation (group Ia showing an increase or no change, and group Ib a decrease).

We next coded the data for Thr308 and Ser473 phosphorylation according to whether they increased (black) or decreased (grey) at *P*<0.05. The coded data are represented within the chart in Figure 3 and are grouped according to the direction of change in Thr308 phosphorylation. Of the 12 cases where Thr308 phosphorylation was increased relative to normal tissue (termed group Ia tumours in Figure 3), Ser473 phosphorylation was increased in parallel in only 6 cases. In three tumours (designated group Ib), Ser473 phosphorylation was regulated in a diametrically opposite manner to Thr308 phosphorylation.

In seven of the eight cases where Thr308 phosphorylation was decreased, Ser473 phosphorylation was decreased in parallel and these are termed group II tumours. In the nine cases where Thr308 phosphorylation was unchanged (*P*>0.05), Ser473 phosphorylation was also unchanged in five but was decreased in the remaining four cases (group III tumours).

To summarise, Akt Thr308 and Ser473 phosphorylation were regulated in an identical manner (increased, decreased or unchanged) in just 18 out of the 29 cases studied. Given the complex interplay between these sites with respect to regulation of kinase activity, this raised the question of which of Akt Thr308 or Ser473 phosphorylation correlates best with Akt activity.

### Evaluation of the phosphorylation of Akt substrates

When active, Akt phosphorylates numerous downstream substrates that bring about the pleiotropic biological effects of this kinase. We undertook a detailed examination of the phosphorylation of three well-characterised Akt substrates in the tumour samples: PRAS40 (on Thr246), TSC2 (on Ser939) and TBC1D4 (on Thr642). PRAS40 and TSC2 are both important regulatory components of the mTOR complex. TBC1D4 is a Rab GTPase-activating protein that plays a role in the regulation of glucose metabolism.

**Figure 4:**
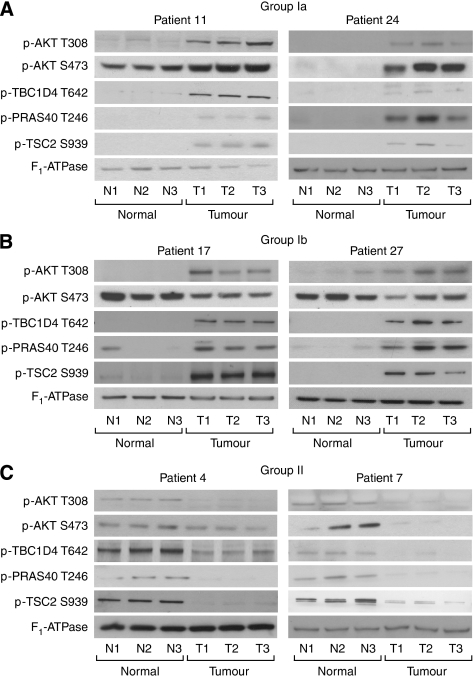
Examples of Akt Thr308 and Ser473 phosphorylation, together with the phosphorylation of downstream substrates. Triplicate lysates of normal (N1–3) and patient-matched tumour (T1–3) tissues were separated on SDS–PAGE gels. Phosphorylation of Akt on Thr308 and Ser473, and of three downstream substrates, was determined by western blotting with phospho-specific antibodies as indicated. An anti-F_1_-ATPase antibody was used as a control for protein loading. (**A**) Examples (patients 11 and 24) of tumours in group Ia. (**B**) Examples of tumours from two patients (patients 17 and 27) in group Ib. (**C**) Examples of two patients (patients 4 and 7) in group II.

The data for the phosphorylation of all three substrates are provided in Supplementary Figures 1–3, and are summarised in the chart of Figure 3. Examples of some of the originating western blot data are provided in Figure 4.

In the nine group Ia tumours, where Akt Thr308 phosphorylation was increased, the phosphorylation of at least one of the three Akt substrates was also increased (Figures 3 and 4A) in all except one patient (patient 18, Figure 3). In all three group Ib tumours, where Akt Thr308 phosphorylation was increased but Ser473 was decreased, the phosphorylation of all three Akt substrates was increased (Figures 3 and 4B), strongly suggesting that Thr308 phosphorylation correlates best with the activity of Akt towards downstream substrates.

In group II tumours, reduced Akt phosphorylation on Thr308 was accompanied by reduced phosphorylation of at least one of the three Akt substrates in all except two tumours (patients 14 and 16; Figures 3 and 4C). Finally, in the nine group III tumours, where Akt Thr308 phosphorylation was unchanged, the phosphorylation of all three Akt substrates was also unchanged in six out of nine patients (Figure 3).

The data in Figure 3 were examined using the Spearman's rank correlation test for pair-wise correlation between the phosphorylation of Akt on Thr308 or Ser473 and the phosphorylation of each of the substrates. The analysis is summarised in Table 1. This demonstrated that the phosphorylation of each of the three Akt substrates correlated with Akt phosphorylation on the activation loop Thr308 (*P*<0.01 in all three cases) but did not correlate with phosphorylation of Ser473 in the hydrophobic motif (*P*⩾0.05 in each case).

In some tumours, phosphorylation of a substrate was observed to increase under conditions where the phosphorylation of Akt and one or both of PRAS40 and TSC2 were unchanged or decreased. This was most specifically the case for TBC1D4 phosphorylation on Thr642 (hence the lower correlation with Akt Thr308 phosphorylation compared with PRAS40 and TSC2). TBC1D4 Thr642 has also been reported to be a substrate for p90 ribosomal S6 kinase 1 and serum- and glucocorticoid-induced protein kinase 1 (Geraghty *et al*, 2007), the activity of which may be elevated in these tumours. This possibility will require future investigation.

## Discussion

In the era of targeted therapy, the assessment of Akt activity in tumours will move from determining its importance in the genesis of cancer to establishing its activity in individual tumours as a means of defining targeted treatment. It is critical that there are methods by which Akt activity can be reliably determined, in the particular tissue and pathological condition being examined or treated.

Lung cancer is the leading cause of cancer death worldwide and NSCLC accounts for 80% of all cases. In NSCLC, Akt activity represents an attractive and quantifiable biomarker for predictive, prognostic and pharmacodynamic purposes (Solomon and Pearson, 2009). The majority of studies aimed at evaluating the predictive power of Akt phosphorylation for patient survival have focussed on Ser473 rather than Thr308 phosphorylation, probably in large part because phosphoSer473 antibodies are relatively effective, particularly in immunohistochemical (IHC) analyses. As a consequence, few studies have analysed Akt phosphorylation on Thr308.

Our data strongly suggest that Akt phosphorylation on Thr308 is a much better indicator of the protein kinase activity of Akt in NSCLC than phosphorylation on Ser473 (Figure 3 and Table 1). We reached this conclusion through examining the correlation between Akt phosphorylation on each of Thr308 and Ser473, and the activity of the Akt kinase as indicated by the state of phosphorylation of three well-characterised Akt substrates: PRAS40, TSC2 and TBC1D4. The observation that Akt Thr308 phosphorylation is the better indicator of activity was most obvious in group Ib tumours in which the phosphorylation of Akt on Thr308 and all three Akt substrates were increased, but the phosphorylation of Akt on Ser473 was decreased (Figures 3 and 4B). However, across all tumours there was a significant correlation between Thr308 phosphorylation and the phosphorylation of all three substrates, but no such correlation for Ser473 phosphorylation.

Our conclusion is consistent with the fact that Thr308 is the major regulator of Akt kinase activity. For example, phosphorylation of purified Akt on Thr308 by recombinant active PDK1 increases Akt protein kinase activity by 30-fold (1, 28, 29), presumably as a result of a major conformational change in the enzyme that allows substrate binding and phosphorylation (Pearce *et al*, 2010). Independent phosphorylation of purified Akt on Ser473 *in vitro* by DNA-dependent protein kinase (DNA-PK) also activates Akt but only by approximately ten-fold (Feng *et al*, 2004). Studies with Akt phosphorylation site mutants suggest that the phosphorylation of Thr308 and Ser473 may act in synergy to stimulate Akt activity (Alessi *et al*, 1996). However, staurosporine attenuates the activation of Akt in cultured HEK293 cells stimulated with insulin in parallel with an inhibition of Thr308 phosphorylation, but has no effect on Ser473 phosphorylation (Hill *et al*, 2001). This observation is consistent with the fact that staurosporine potently inhibits PDK1 (32) but has little effect on mTORC2 (Hresko and Mueckler, 2005), which is widely agreed to be the physiologically relevant Ser473 kinase in most cell types (Sarbassov *et al*, 2005). Taken together, the data suggest that Ser473 does not play a significant role in regulating Akt activity in HEK293 cells and our results strongly suggest that Thr308 phosphorylation is the primary regulator of Akt activity, at least in NSCLC tumours.

That we demonstrate Thr308 phosphorylation to be a better marker for Akt activity than Ser473 phosphorylation could help to explain why Tsurutani *et al* (2006) found that Thr308 phosphorylation was a better predictor than Ser473 phosphorylation for poor overall survival in NSCLC. We propose that this was because these tumours possess the highest Akt activity, which was responsible for driving tumour cell proliferation and survival. As Ser473 is phosphorylated by the mTORC2 complex (and possibly integrin-linked kinase and DNA-PK in some cell types), measurements of its phosphorylation may report the activity of the upstream Ser473 kinase(s) rather than activity of Akt itself, and hence care should be exercised when using this phosphorylation site as a biomarker.

To our knowledge, no studies have assessed Akt activity in NSCLC by looking at phosphorylation of both Ser473 and Thr308 in addition to substrate phosphorylation. Although when studying Akt activation in NSCLC, Balsara *et al* (2004) found that the phosphorylation of two Akt substrates (mTOR and FKHR) was significantly associated with the phosphorylation of Akt on Ser473. There are notable differences in methodology between this study and ours that may explain the discrepancy in results with respect to the correlation of Ser473 with substrate phosphorylation. By rapidly flash freezing tissue samples, the current study was designed to minimise changes in phosphorylation whereas Balsara *et al* (2004) used archival paraffin-embedded samples that may not have been subject to such handling. Secondly, we obtained our data by a comparison of phosphorylation in tumour tissue with that in patient-matched normal tissue rather than correlating Akt and substrate phosphorylation in individual tumour sections.

A recent study reported that the phosphorylation of PRAS40 on Thr246 positively correlated with the activation of the PI3-kinase pathway and predicted an increased sensitivity of tumour cell growth to an Akt inhibitor in lung and breast cancer cell lines (Andersen *et al*, 2010). Taken together with our own data, showing PRAS40 to strongly correlate with Akt Thr308 phosphorylation, suggests that Akt Thr308 and/or PRAS40 Thr246 phosphorylation would be good candidate biomarkers to assess Akt activity in NSCLC.

The data in our current study suggest that the regulation of Akt activity in NSCLC is complex. Akt activity, as judged by increased phosphorylation of Akt on Thr308 and at least one downstream substrate, was elevated in 12 (41%) of the tumours we examined. We did not examine the upstream defects responsible for the activation of Akt; however, we did find that EGFR tyrosine phosphorylation was significantly elevated in 6 of these 12 tumours, suggesting that changes in EGFR activity might have been responsible (data not shown). Clearly, however, other mechanisms will also play a role such as increased activity of the insulin-like growth factor receptor or cMet and PTEN mutation status.

It is of interest that Akt Thr308 phosphorylation and activity were reduced in a significant number of tumours (group II; Figure 3). This may be a result of feedback inhibition of Akt because of persistent activation of the downstream mTOR complex, as previously reported (Shah *et al*, 2004; O’Reilly *et al*, 2006). Therefore, we propose that this group of tumours would be unlikely to respond to inhibitors of Akt and PI3-kinase.

Our observations have important consequences for the future use of Akt as a molecular biomarker in predicting the efficacy of targeted therapy against the PI3-kinase/Akt pathway and its regulators. We propose that any evaluation of the link between Akt phosphorylation in tumour samples and the prediction or prognosis of disease should focus on the phosphorylation of Thr308 and/or at least one downstream Akt substrate, rather than Ser473 phosphorylation alone, in order to more reliably determine the activity of the kinase and thus better guide disease prognosis and treatment. Furthermore, although our current results pertain to NSCLC, they almost certainly have relevance to other tumour types in which this kinase plays a role in tumourigenesis.

## Figures and Tables

**Table 1:** Correlation between the phosphorylation of Akt on Thr308 or Ser473 and the phosphorylation of downstream Akt substrates

	**pPRAS40**	**pTSC2**	**pTBC1D4**
*Akt-pThr308*			
*R*	0.752	0.676	0.465
*p*	<0.1 × 10^–3^	<0.1 × 10^–3^	8.3 × 10^–3^
			
Akt-*pSer473*			
*R*	0.352	0.229	0.137
*p*	0.052	0.216	0.462

Abbreviations: Ser473=serine 473; Thr308=threonine 308.

The table provides the correlation coefficient (*R*) and degree of significance (*p*) obtained for each variable shown using the Spearman's pair-wise correlation test.
