# Supplementary figures and images for: Akt phosphorylation on Thr308 but not on Ser473 correlates with Akt protein kinase activity in human non-small cell lung cancer
Source: Br J Cancer. 2011 Apr 19;104(11):1755–61. doi: 10.1038/bjc.2011.132 (PMC3111153; doi:10.1038/bjc.2011.132)

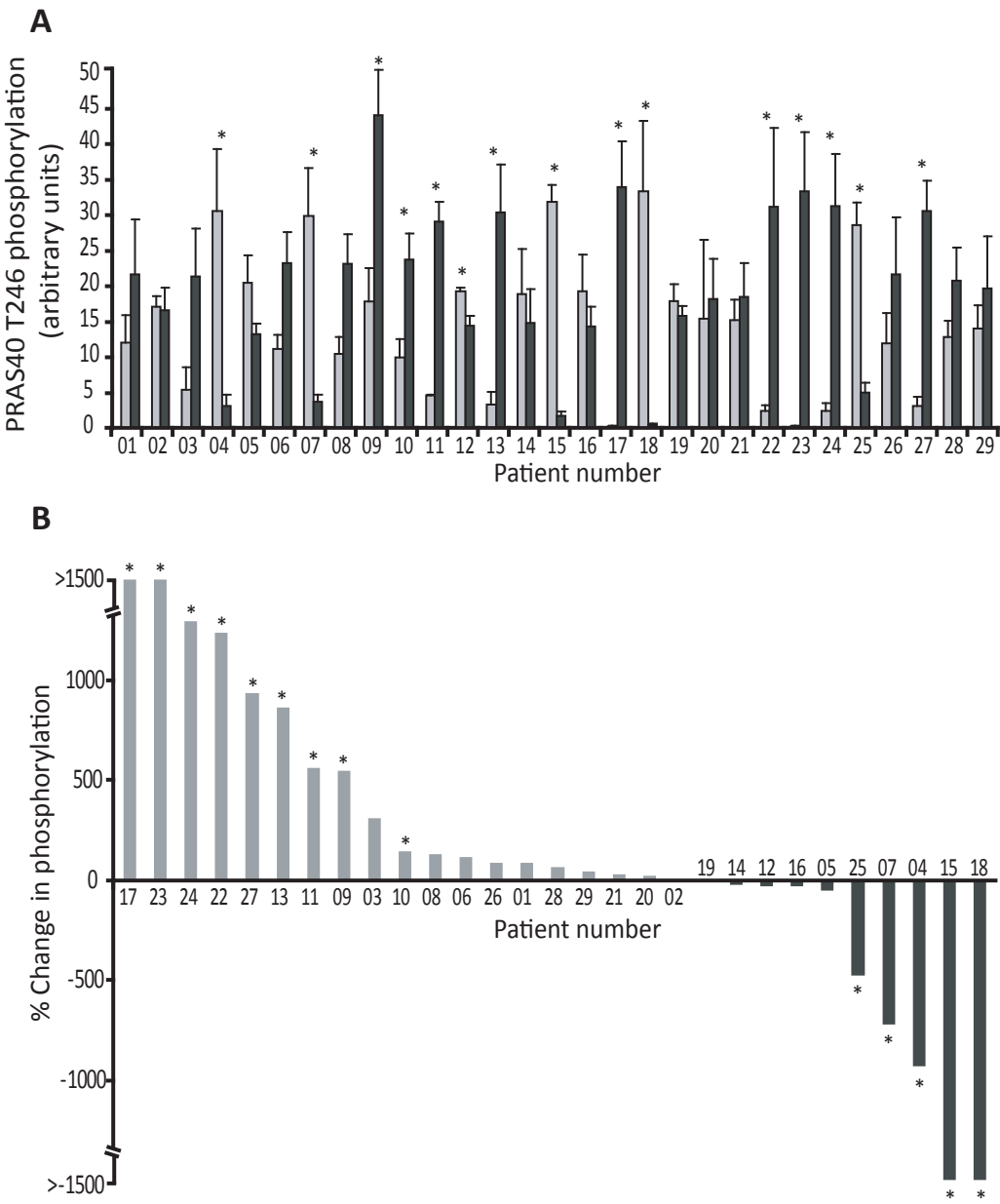

Supplement: Supplementary Figure 1 [file bjc2011132x1.pdf]

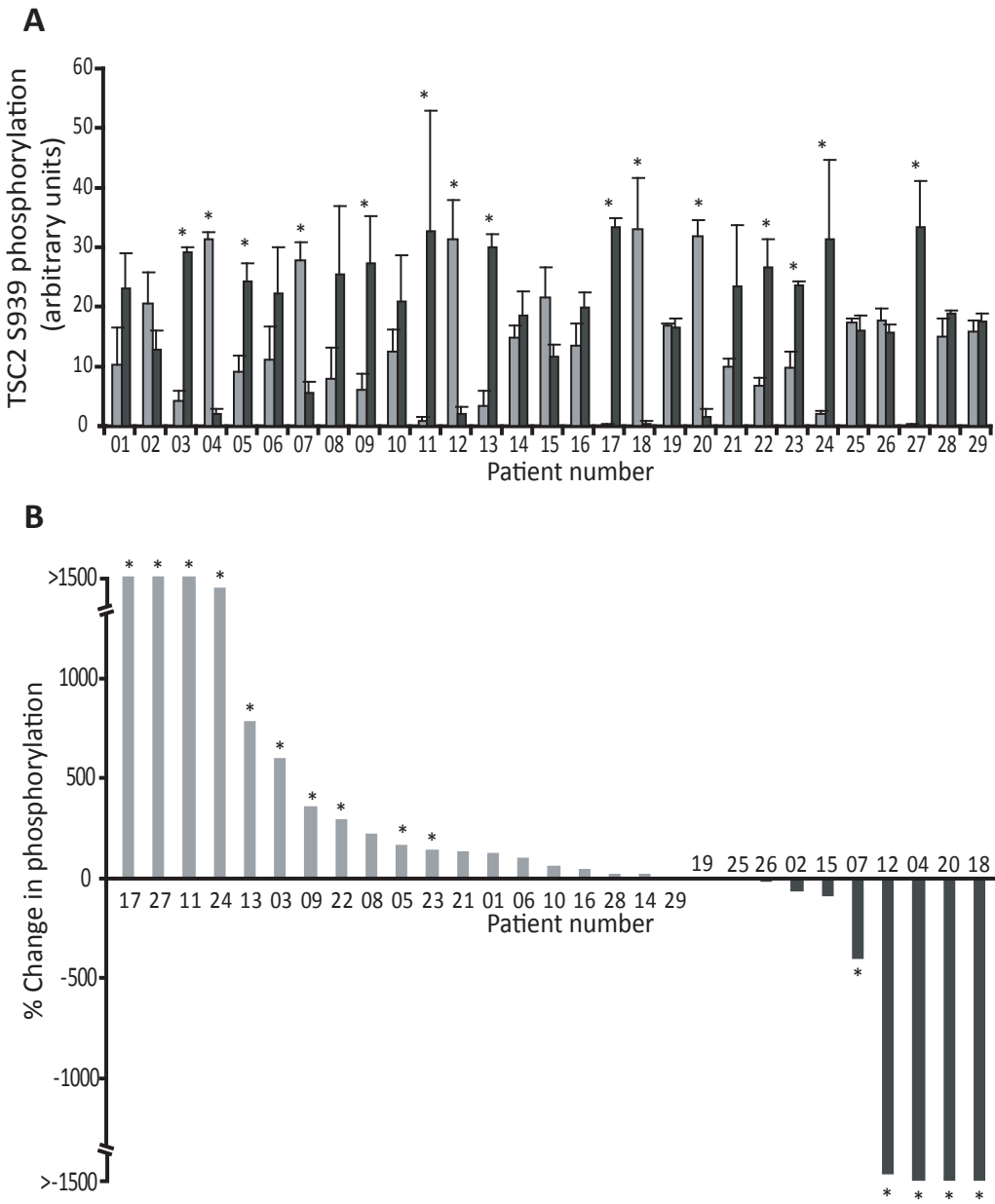

Supplement: Supplementary Figure 2 [file bjc2011132x2.pdf]

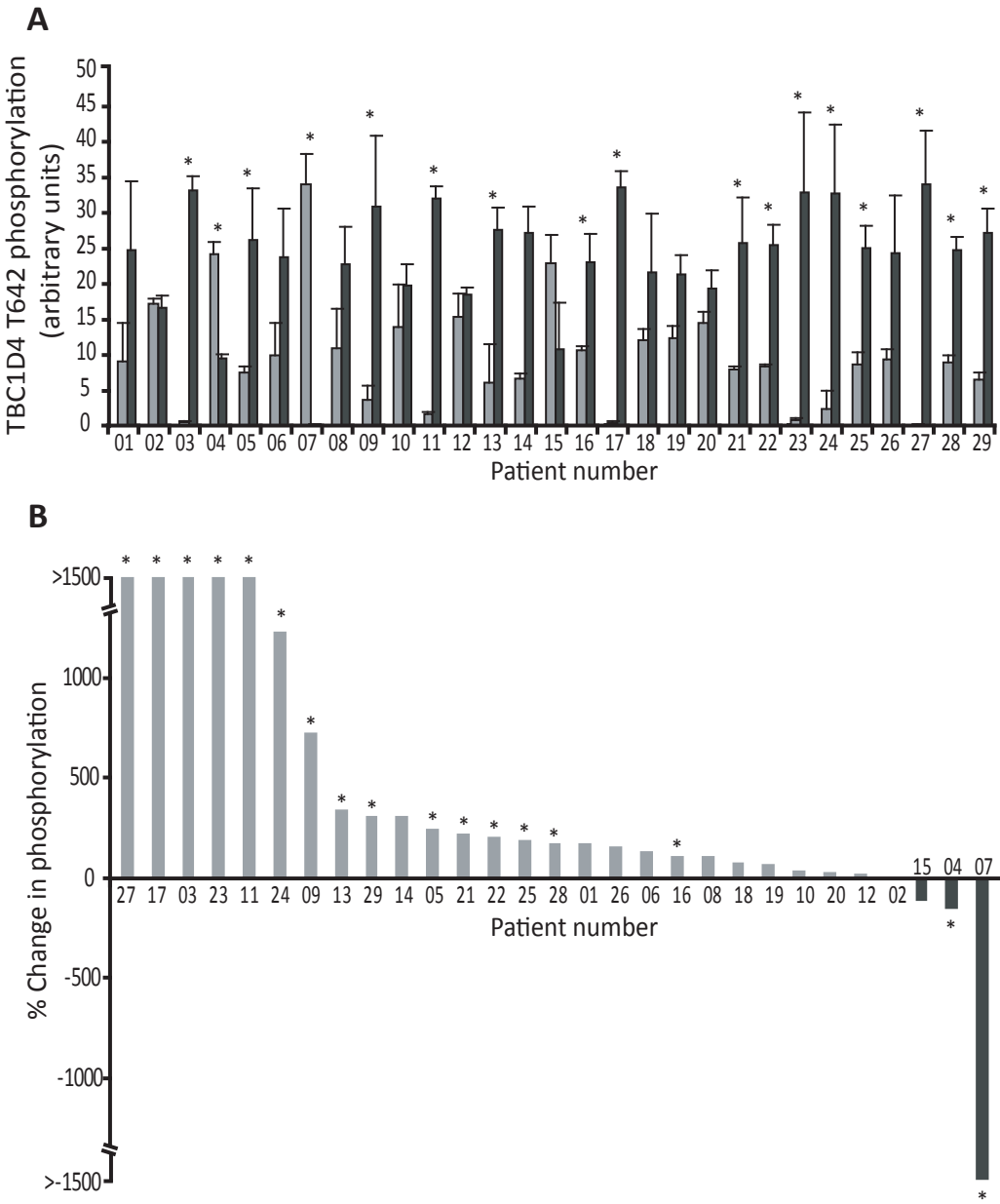

Supplement: Supplementary Figure 3 [file bjc2011132x3.pdf]
