# Supplementary material for: Akt phosphorylation on Thr308 but not on Ser473 correlates with Akt protein kinase activity in human non-small cell lung cancer
Source: Br J Cancer. 2011 Apr 19;104(11):1755–61. doi: 10.1038/bjc.2011.132 (PMC3111153; doi:10.1038/bjc.2011.132)
Supplement: Supplementary Figure Legends [file bjc2011132x4.doc]

**Supplementary Figure legends.**

**Supplementary Figure 1 Phosphorylation of PRAS40 on Thr246 in NSCLC tumour tissue in comparison to patient matched normal lung tissue.**

Triplicate samples of lysate from patient-matched normal (N1-3) and tumour (T1-3) tissues were separated on SDS-PAGE gels. Phosphorylation of PRAS40 on Thr246 was determined by western blotting with a pPRAS40-Thr246 antibody followed by quantitation by densitometric scanning. A: Each bar represents the mean phosphorylation for normal (N1-3; light grey) or tumour (T1-3; black) tissues for each patient (mean ± SEM). The strength of evidence for a difference in phosphorylation between the normal and tumour samples was determined by a Kruskal-Wallis test
(*indicates p<0.05). B: Shows the percentage change in PRAS-Thr308 phosphorylation with patients ranked in order of the extent of the percentage change.

**Supplementary Figure 2 Phosphorylation of TSC2 on Ser939 in NSCLC tumour tissue in comparison to patient matched normal lung tissue.**

Identical to the data in Supplementary Figure 1 except that TSC2 phosphorylation on Ser939 was examined.

**Supplementary Figure 3 Phosphorylation of TBC1D4 on Thr642 in NSCLC tumour tissue in comparison to patient matched normal lung tissue.**

Identical to the data in Supplementary Figure 1 except that TBC1D4 phosphorylation on Thr642 was examined.
